# Optimizing EEG Signal Integrity: A Comprehensive Guide to Ocular Artifact Correction

**DOI:** 10.3390/bioengineering11101018

**Published:** 2024-10-12

**Authors:** Vincenzo Ronca, Rossella Capotorto, Gianluca Di Flumeri, Andrea Giorgi, Alessia Vozzi, Daniele Germano, Valerio Di Virgilio, Gianluca Borghini, Giulia Cartocci, Dario Rossi, Bianca M. S. Inguscio, Fabio Babiloni, Pietro Aricò

**Affiliations:** 1 Department of Computer, Control, and Management Engineering, Sapienza University of Rome, 00185 Roma, Italy; daniele.germano@uniroma1.it (D.G.); valerio.divirgilio@uniroma1.it (V.D.V.); biancams.inguscio@uniroma1.it (B.M.S.I.); or pietro.arico@brainsigns.com (P.A.); 2 BrainSigns S.r.l., Industrial Neurosciences Lab, 00198 Rome, Italy; gianluca.diflumeri@uniroma1.it (G.D.F.); andrea.giorgi@uniroma1.it (A.G.); alessia.vozzi@uniroma1.it (A.V.); gianluca.borghini@uniroma1.it (G.B.); giulia.cartocci@uniroma1.it (G.C.); fabio.babiloni@uniroma1.it (F.B.); 3 Department of Anatomical, Histological, Forensic and Orthopaedic Sciences, Sapienza University of Rome, 00185 Roma, Italy; rossella.capotorto@uniroma1.it; 4 Department of Molecular Medicine, Sapienza University of Rome, 00185 Roma, Italy; dario.rossi@uniroma1.it; 5 Department of Physiology and Pharmacology “Vittorio Erspamer”, Sapienza University of Rome, Piazzale Aldo Moro 5, 00185 Rome, Italy; 6 College of Computer Science and Technology, Hangzhou Dianzi University, Hangzhou 310005, China

**Keywords:** EEG, signal processing, ocular artifacts

## Abstract

Ocular artifacts, including blinks and saccades, pose significant challenges in the analysis of electroencephalographic (EEG) data, often obscuring crucial neural signals. This tutorial provides a comprehensive guide to the most effective methods for correcting these artifacts, with a focus on algorithms designed for both laboratory and real-world settings. We review traditional approaches, such as regression-based techniques and Independent Component Analysis (ICA), alongside more advanced methods like Artifact Subspace Reconstruction (ASR) and deep learning-based algorithms. Through detailed step-by-step instructions and comparative analysis, this tutorial equips researchers with the tools necessary to maintain the integrity of EEG data, ensuring accurate and reliable results in neurophysiological studies. The strategies discussed are particularly relevant for wearable EEG systems and real-time applications, reflecting the growing demand for robust and adaptable solutions in applied neuroscience.

## 1. Introduction

The electroencephalographic (EEG) signal is one of the most informative electrophysiological biosignals, used across various research areas and biomedical applications, including brain–computer interfaces (BCIs), mental state assessments, neurofeedback, and more. Recent advancements in the development of more effective wearable EEG devices equipped with fewer sensors have enabled their use outside research laboratories. For instance, EEG-based BCI systems operate based on outputs derived from brain activity, either with voluntary control (active BCIs) or without it (passive BCIs, [1,2]), to facilitate communication through ongoing mental or emotional states while the user engages in other tasks. These systems depend on analyzing and interpreting EEG signals through specific time and frequency domain features.

**Figure 1:**
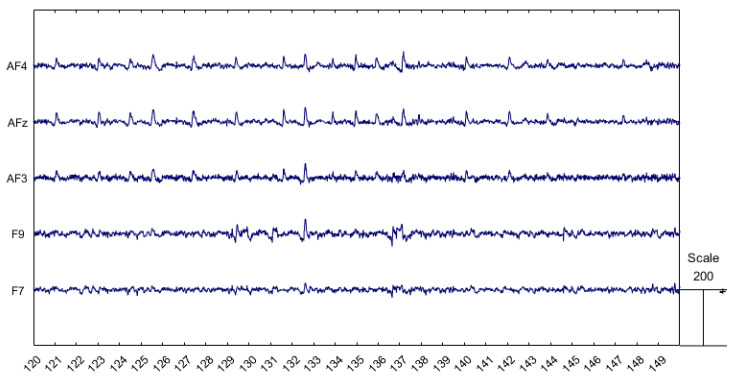
Raw EEG signal on frontal electrodes showing ocular artifacts, which can be easily identified due to their larger amplitudes compared to the EEG signal.

However, EEG-based features are challenged by a low signal-to-noise ratio, and several confounding factors can distort or obscure the desired physiological information. Among these, artifacts generated by ocular movements (such as eyeblinks and saccades) are particularly significant due to three main reasons:

- The power spectrum of ocular movements overwhelms informative EEG-related features, as their bandwidth (3–15 Hz) overlaps with the frequency range of important neurophysiological contents like the EEG theta and alpha bands. Ocular artifacts can also interfere with time-domain analyses, such as the extraction of Evoked Potentials [3].
- The frequency of ocular artifacts is too high to simply remove affected EEG epochs, with occurrences ranging from 12 to 18 blinks per minute.
- Ocular artifacts are also characterized by much larger amplitudes compared to normal EEG signals, as shown in Figure 1. This makes them more easily identifiable, allowing expert operators to detect them visually and enabling algorithms to recognize them automatically. However, if not properly addressed, these artifacts can substantially distort the EEG signal, potentially leading to misinterpretations.

This tutorial focuses on methodologies to address this specific category of EEG signal artifacts, i.e., those caused by eye movements, especially ocular blinks, by correcting them without losing informative neurophysiological data. Ocular blinks contaminate the EEG signal due to major physiological sources: the corneo-retinal dipole, eyelid movements, and extraocular muscles. The corneo-retinal dipole represents the positive charge of the cornea relative to the retina, causing potential changes at EEG sensors during eyeball rotation. Eyelid movements, more pronounced during blinks, introduce high-amplitude potential field changes, and contractions of extraocular muscles affect EEG signal amplitude. Notably, within the 3–15 Hz frequency range, artifacts from the corneo-retinal dipole and eyelid movements are prominent.

As previously mentioned, these artifacts cannot be simply removed by discarding the affected EEG segments, as this would result in a significant loss of neurophysiological content associated to the processed signal. Therefore, it appears to be essential to correct EEG segments containing ocular blink artifacts by purging the contributions of these artifacts.

Therefore, the present tutorial aims at providing a comprehensive framework describing the paramount EEG signal-processing techniques for the identification and correction of ocular blink artifacts.

## 2. General Framework

As mentioned within the Introduction section, ocular blink artifacts are generated by the movement of the eyelids, leading to significant changes in the EEG signal. These artifacts are characterized by high-amplitude spikes and primarily affect the EEG signal within the 3–15 Hz frequency range. This range overlaps with important brain rhythms like theta and alpha bands, making it crucial to address these artifacts for accurate data interpretation.

The state of the art in the context of the EEG signal processing for identifying and correcting ocular blink artifacts shows different kind of techniques, each of which is specifically indicated for specific cases. Besides the selection of an approach, an aspect which will be addressed in the following sections, the common operations required for the correct identification and correction of the ocular blink artifacts include preprocessing the EEG signal. Therefore, it is indicated to apply a band-pass filter to eliminate low-frequency drifts and high-frequency noise from the data. Optionally, re-referencing the data to a common average or a specific reference electrode might help to minimize noise and enhance signal quality.

Subsequently, different techniques were validated by the scientific literature for the identification and correction of ocular blink artifacts. In this regard, a first discrimination must be performed according to the EEG channel number employed for the signal collection. If the channel number results to be consistently high (e.g., above 40), previous works have demonstrated that an Independent Component Analysis (ICA) might be the best approach for identifying the ocular blink components in order to finally remove them without negatively affecting the neurophysiological content of the signal. In recent years, various other techniques have been successfully explored and validated for the correction of ocular blink artifacts from EEGs. The following list includes the main categories of the most employed and transversally validated techniques:

- **Regression-based methods**: These correspond to an approach requiring an ocular blink template, which is specific to each subject. The several regression-based methods proposed in the scientific literature rely on electrooculography (EOG) or, in case of unavailability of the EOG channel, on frontal, prefrontal, and anterofrontal EEG channels as ocular blink templates. Such methods generally require a calibration run specifically corresponding to the ocular blink template collection. Within the state of the art, these methods can be divided into linear regression-based methods, i.e., methods which foresee the modeling and subtraction of the ocular blinks’ contribution using the ocular blink template as a regressor, and adaptive filtering-based methods, i.e., methods which foresee the adjustment of dynamic model parameters for a better fit with the EEG signal to be corrected.
- **Independent Component Analysis (ICA)**: This includes a wide range of methodologies based on the decomposition of the EEG signal to its independent components. Therefore, this approach foresees independent component analysis by identifying the ones associated with the ocular blinks and removing them.
- **Artifact Subspace Reconstruction (ASR)**: This is an advanced technique that operates by detecting and reconstructing the subspace of the EEG data contaminated by artifacts. This method leverages the statistical properties of the EEG signal to differentiate between neural activity and artifacts. By identifying the subspace where artifacts dominate, ASR can reconstruct the clean EEG signal by removing the contributions from this subspace.
- **Deep learning-based algorithms** [4,5,6]: These correspond to a new branch of methods that relies on deep neural networks. Networks can be trained on clean EEG signals and learn to recognize non-physiological patterns in EEG signals and correct them.

## 3. Methods and Principles

Once the general framework is defined, including the main categories of signal processing techniques for identifying and correcting ocular blink artifacts from EEGs, the present section provides a comprehensive description of each of state-of-the-art method.

### 3.1. Regression-Based Methods

**Figure 2:**
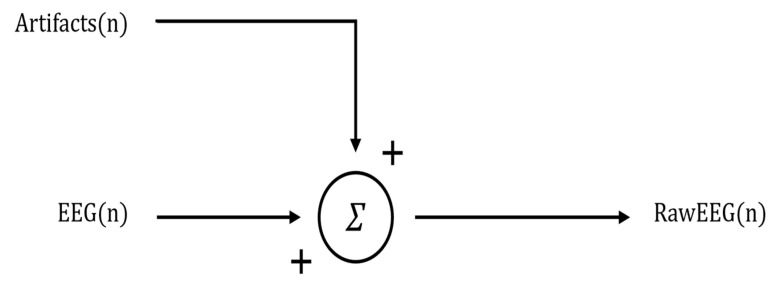
Signal composition block diagram.

Regression-based methods are the simplest and most traditional methods for removing ocular artifacts from EEG signals [7,8,9]. These methods are applied under a linearity assumption, that is, the assumption that each signal is the cumulative sum of the brain signal and artifacts. Thus, the total signal can be described as the linear and time-invariant combination of these contributions, as illustrated by Formula (1) and Figure 2 [7,10].

RawEEG(n) = EEG(n) + artifacts(n),(1)

where RawEEG is the signal recorded by the electrodes, EEG is the desired cleaned signal, and artifacts are other non-target signals that contaminate the EEG signal. Therefore, it is evident that the target EEG signal cleaned of artifacts can be derived by subtracting the artifacts from the RawEEG if information and/or estimation of artifacts is available.

For this reason, these methods are suitable for correcting ocular artifacts, since it is usually possible to estimate the pattern of ocular artifacts, for example, by using electrooculographic channels.

It is important to note that the influence of ocular artifacts on EEG signals is variable, and this variability depends on the position of the recording electrodes. In fact, electrodes closer to the eye location, such as the frontal ones, will be more affected by artifact influence. Thus, more specifically, Formula (1) can be written as Formula (2) [11].

(2) $$RawEEG_{e_{i}}\left(n\right)=EEG_{e_{i}}\left(n\right)+\beta_{e_{i}}artifacts\left(n\right),$$

where $e_{i}$ represents the i-th electrode and $\beta_{e_{i}}$ is the weight associated with the i-th electrode; thus, the correction through regression needs to be made on each channel independently.

The first and simplest form of a regression-based algorithm to remove EOG artifacts from EEG signals was proposed by Hilyard and Galambos [8] using a pre-experimental calibration run, in which the participants were asked to voluntary produce ocular artifacts, as the EOG template. However, in the following years, it has been demonstrated that spontaneous and imposed eye movements are different [12]. Consequently, the method evolved to require that the calibration signal be acquired during the same session as the EEG recording. Following this advancement, two main regression-based methods have been proposed and validated in the scientific literature: time domain [12,13] and frequency domain regression [14]. Both methods exploit preliminary calibration tasks to estimate $\beta_{ei}$ coefficients (regression phase) for each of the EEG channels [7], but collect spontaneous blinking activity through the EOG channel. Then, in the correction phase, the EOG component weighted by the specific previously estimated $\beta_{ei}$ is removed by subtraction from each EEG signal. Both the time and frequency domain approaches appear to result in similar performances, even though these two methods strongly differ in how they estimate the ocular interference degree on the EEG signal (i.e., the estimation of the weights coefficient, which is performed in either the time or frequency domain depending on the method). This similarity encourages the use of the time domain method, as it is the simpler of the two [14].

Thus, in the following tutorial, only the time domain regression method will be further analyzed. In particular, the following processing chain will refer to the Gratton and Cole algorithm [12] that is still used today. Additionally, this algorithm is used as a base for some multi-stage algorithms that rely on regression [15,16].

**Figure 3:**
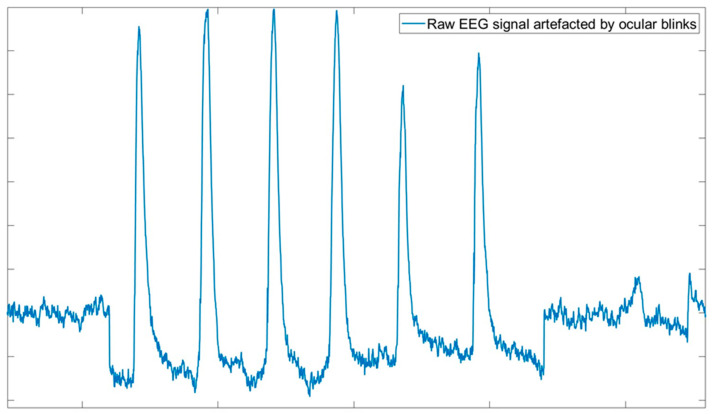
Raw EEG signal affected by ocular artifacts. Such artifacts can be easily visually recognized as the prominent peaks visible along the signal trace.

**Figure 4:**
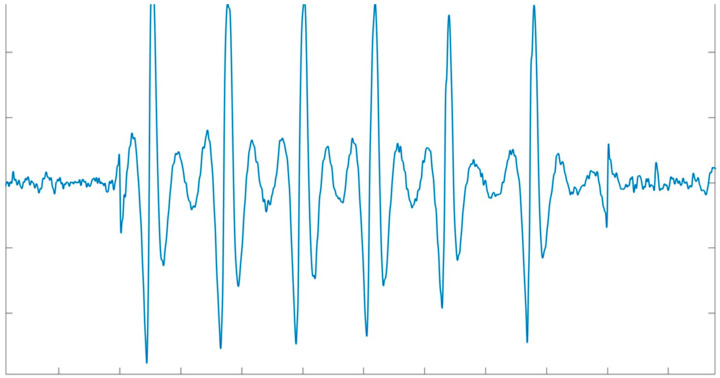
Example of artifactual component derived from the EEG signal affected by ocular artifacts through the regression-based algorithm.

The computational procedure will refer to a single channel and needs to be iterated across channels:

1. The rawEEG signal is typically filtered to pass only interesting frequencies (for example, between 1 and 50 Hz) to eliminate slow fluctuations and high-frequency disturbances in order to remove artifacts whose frequencies do not overlap with the EEG spectrum.
2. The EOG signal is low-pass filtered (cut-off frequency 15 Hz) to eliminate high-frequency ailments and to increase method accuracy, since it has been demonstrated that the main spectral content of ocular blinks is up to 15 Hz (Figure 3).
3. Temporal alignment and segmentation of EEG and EOG signals is conducted, which constitute a preliminary step necessary in order to accurately correct ocular artifact contributions in EEG signals.
4. $\beta_{e_{i}}$ coefficient estimation is the most important step of the regression algorithm. In fact, once these coefficients are estimated, the algorithm can be considered calibrated and the EEG signal can also be correct in real time. This step is composed of three sub-steps:
  - The raw EEG and raw EOG signals are firstly averaged across epochs; these averages represent the “signal baseline” [17].
  - The averages evaluated in the previous step are then subtracted from each epoch of the EEG and EOG signals, respectively. After this subtraction, the resulting signals represent the “deviation from the baseline”.
  - Then, the “deviation from the baseline” signals serve as variables for the correlation analysis. The correlation is computed considering the EOG as the independent variable and the EEG as the dependent variable. Finally, the correlation coefficient is the estimation of $\beta_{e_{i}}$ (Figure 4).

**Figure 5:**
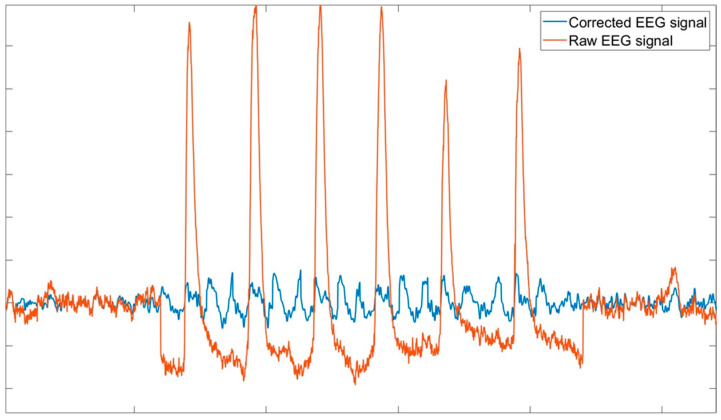
Overlapped representation of the raw (orange line) and clean (blue line) EEG signals. The figure shows how the algorithm successfully identified and corrected the ocular artifacts.

Once the weights associated with each electrode are estimated, the EEG signal cleaned up of artifacts can be derived by subtracting the artifacts from the RawEEG (Figure 5).

**Figure 6:**
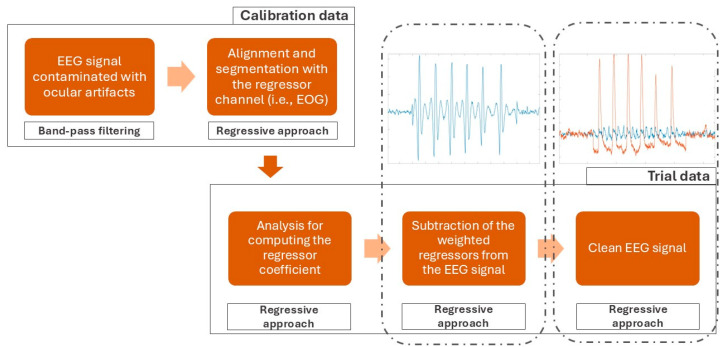
Block diagram of the principal steps for approaching the identification and correction of ocular artifacts from an EEG signal through a regression-based method.

Following the presented steps, this algorithm can be easily implemented on Phyton (v 3.12.4, Python Software Foundation License) or MATLAB (v2024, MathWorks, Natick, MA, USA) using an already existing open-source library and toolbox, such as mne.preprocessing [18] on Phyton and EEGLAB (v2024.2, Swartz Center for Computational Neuroscience, San Diego, CA, USA) on MATLAB. The following diagram block (Figure 6) outlines the principal steps for approaching the identification and correction of ocular artifacts from an EEG signal through a regression-based method.

Among the most recent regression-based methods proposed in the scientific literature, the o-CLEAN method appears to be among the most promising [19]. Such an algorithm combines a regression-based approach [16] and an adaptive filtering technique [20] for identifying and correcting ocular artifacts from EEG signals. This method is ideal for transversal application in the context of the EEG processing, and it is indicated to be applied when the EEG signal is collected in highly controlled environments, with high-density EEG equipment, and within out-of-the-lab applications.

### 3.2. Independent Component Analysis (ICA)

The ICA is a widely used signal processing technique for separating mixed signals into their independent sources. In the context of EEG signal processing, ICA is particularly effective for identifying and removing ocular blink artifacts. This section of the tutorial will provide a detailed description of the ICA method for ocular blink artifact correction in EEG signals. The method aims at decomposing the EEG signal into its independent components. The ICA relies on the assumption for which the observed EEG signals are linear mixtures of statistically independent source signals, including neural activity and artifacts. By leveraging this assumption, ICA can separate the sources and isolate the ocular artifacts for removal.

**Figure 7:**
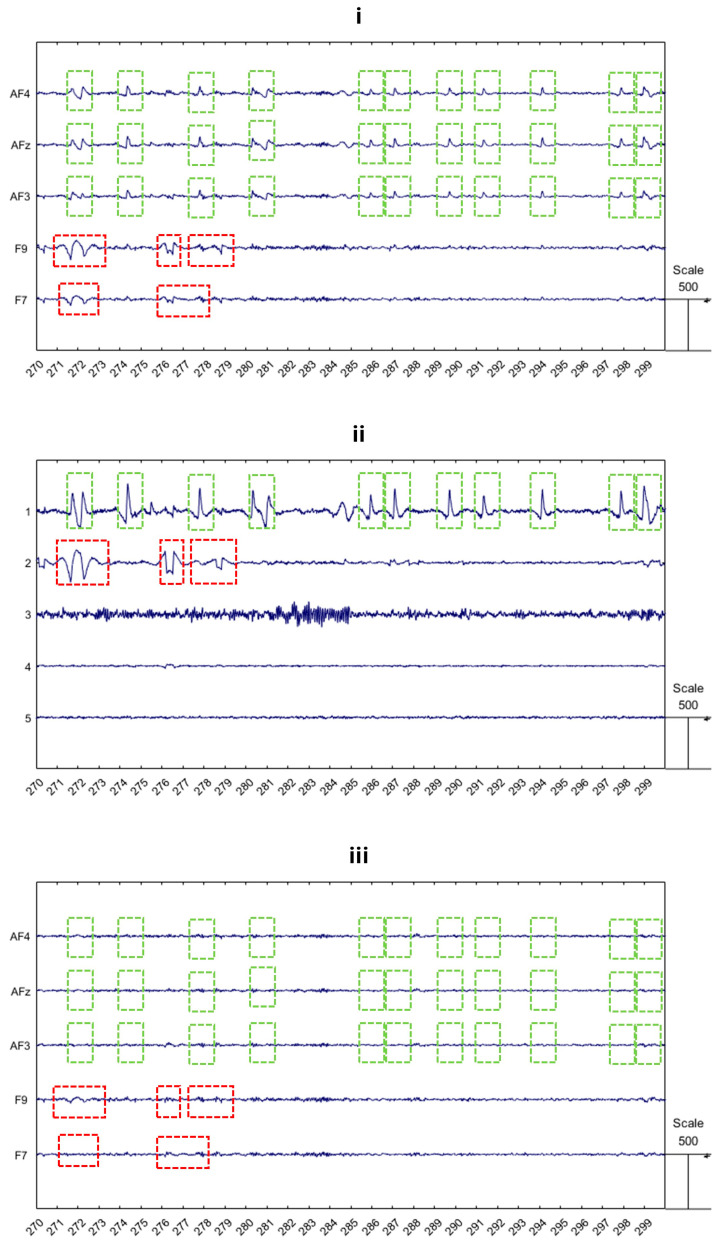
Example of ICA’s performance in removing ocular artifacts. The presented plots show: (**i**) the raw EEG from frontal electrodes; (**ii**) the first five components from ICA, ordered by energy; and (**iii**) the clean EEG from the same electrodes after removing the artifactual components (specifically, the first and second components). Green rectangles highlight blink patterns in both the raw EEG and the ICA components, while red rectangles indicate saccade patterns. After cleaning the EEG signal, these rectangles no longer contain artifact patterns, demonstrating the effectiveness of the artifact removal process.

Within the scientific literature, different ICA-based approaches have been proposed and validated. Among the most recent and high-performing ones, the Adaptive Mixture ICA [21] appears to be of particular interest since it does not strictly require EOG channels, even if its efficiency consistently depends on the available EEG channel number. Therefore, the following processing chain relates to the AMICA, but it can be easily generalized to the processing chain associated with other recent ICA-based techniques:

1. The raw EEG signal is often band-pass filtered (e.g., 1–50 Hz) to remove slow drifts and high-frequency noise. This step ensures that the signal primarily contains frequencies of interest, removing artifacts that do not overlap with the EEG spectrum.
2. The EEG signal can be optionally segmented in epochs of a specific time duration. This step is especially indicated in the case of time-locked EEG feature computation (e.g., event-related potentials).
3. Decomposition of the EEG signal occurs through the estimation of a mixing matrix A, and a source matrix, S, such that X = AS, where X corresponds to the observed EEG signal [22,23].
4. The step corresponding to the independent components analysis is the most relevant within the ICA application. In fact, the independent components of the EEG signal must be analyzed for identifying those that correspond to the ocular blink contribution. In this regard, two approaches have been extensively observed and validated by previous scientific works:
  - The components are visually inspected for the purpose of identifying which of them are related to the ocular blink artifacts. For this approach, an expert operator is required to perform the signal processing, who must be able to correctly recognize visual ocular blink patterns.
  - Automated methods can also be used, such as correlation with electrooculogram (EOG) signals, kurtosis, or power spectral density analysis [24].
5. Once the ocular blink components are identified, such components must be removed.
6. Finally, the clean EEG signal, X_clean, is reconstructed by multiplying the mixing matrix A with the modified source matrix S_source (the matrix S without the independent components associated with the ocular artifacts) [23,25] (Figure 7).

Following the described steps, ICA can be implemented in Python or MATLAB using existing open-source resources, i.e., EEGLAB on MATLAB and *mne.preprocessing* on Python [18]. It is important to note that, especially for ICA, there are various methods for performing ICA, such as SOBI, AMICA, and RunAMICA, each of which offers different capabilities to separate components, balancing precision with computational cost. The choice of a method for the initial decomposition phase should be made with careful consideration of the specific requirements of the analysis.

### 3.3. Artifact Subspace Reconstruction (ASR)

Artifact Subspace Reconstruction (ASR) is a modern and advanced method for removing artifacts from EEG signals. Similarly to ICA, ASR employs a sophisticated approach, decomposing the signal into a subspace that separates the artifacts from the neural activity. This method involves detecting and eliminating components associated with artifacts by employing a statistical model to differentiate between brain signals and ocular interference. The method can be theoretically described as follows [26,27,28]:

(3) X=MS,

(4) $$Y=V^{T}X=V^{T}MS,$$

(5) $$S_{clean}={\left({V_{trunc}}^{T}M\right)}^{+}Y,$$

(6) $$X_{clean}=MS_{clean},$$

where X is the uncleaned EEG signal and S is the ASR’s sources signal matrix. M is defined by ASR’s method as the mixing matrix and is calculated as the square root of the covariance matrix of the calibration data. V represents the eigenvector matrix obtained from the decomposition of M. Thus, Y is the projection of the uncleaned EEG signal onto the component space. The core idea is that S can be reconstructed using the truncated version of the matrix V, which retains only the non-rejected principal components.

The rejection of principal components is based on a threshold cut-off for the standard deviation (SD) evaluated on each of the components of the calibration data (i.e.,$\;Y_{c}=V^{T}X_{c}$). Typically, the recommended SD threshold for principal components ranges from 10 to 30 SDs [26,27,28,29]. Therefore, all the principal components whose SD is above the respective threshold value are truncated from the V matrix.

The first ASR’s processing chain, presented by Khote [30] was designed for an online application. However, future changes also allowed for offline signal cleaning [26]. These two implementations differ only in the calibration phase and will both be described in the following chain:

1. The raw EEG signal is preliminary band-pass filtered to remove both high-frequency and low-frequency noise (1–50 Hz). This step ensures that the signal mainly includes only the artifacts that overlap the EEG spectrum in order to improve ASR accuracy.
2. The ASR algorithm is calibrated using the reference data, which should be free from artifacts. As presented before, this step may differ depending on which version of the algorithm is chosen.
  - For online applications, it is essential to acquire reference data before the experiment during a calibration run, which typically consists of 1–2 min of EEG recording with eyes closed. Although 1–2 min is the recommended duration for optimal algorithm calibration, even 30 s can be sufficient. It is crucial that the algorithm is calibrated for each subject using their respective calibration run to ensure accuracy and effectiveness.
  - For offline applications, ASR can automatically extract artifact-free portions from the EEG signal (i.e., not from the calibration run) and concatenate them to create 1–2 min of “calibration” data.
3. In order to determine the threshold, ASR firstly computes the mixing matrix $M_{c}$ from the calibration data. Next, the matrix $V_{c}$ is obtained using Singular Value Decomposition (SVD) from the mixing matrix. Then, the reference EEG data are projected into the principal component space using Formula 4. The principal components (*Y*) are segmented into 0.5 s windows, and the mean (*μ*) and standard deviation (*σ*) are evaluated across the windows for each component. Finally, the threshold $\Gamma_{i}=\mu_{i}+k\sigma_{i}$ is defined for each i-th component. As discussed above, the usual value of parameter k is between 10 and 30.

**Figure 8:**
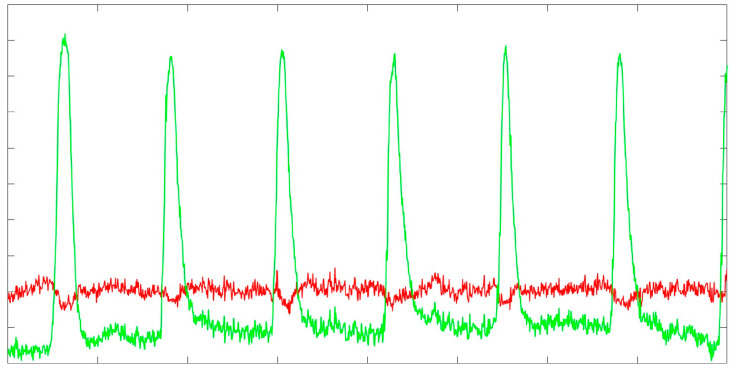
Representation of the ASR method performance for correcting ocular blink artifacts from the EEG signal. The figure shows how the method was effective in identifying and correcting the ocular artifacts from the raw EEG signal (green line) and obtaining the clean (red line) EEG trace.

In the last step, the clean EEG is reconstructed using Formula (5) and Formula (6). The mixing matrix M for the EEG signal is evaluated similarly to the calibration data, and SVD is used to extract V. Finally, the $V_{trunc}$ is obtained by rejecting from V all the components whose variance (i.e., eigenvalue associated with the PCs) is larger than the rejection threshold evaluated in step 3, which was projected from $V_{c}$ to V (Figure 8).

By following the outlined steps, this algorithm can be readily implemented in Python or MATLAB using existing open-source libraries and toolboxes, such as EEGLAB’s plugin *clean_rawdata* on MATLAB and ASRpy on Phyton [31,32].

### 3.4. Deep Learning-Based Algorithms

Deep learning-based algorithms have gained increasing popularity in recent years. This growing interest is driven by advancements in computational power, the availability of larger datasets, and the development of new network architectures and learning techniques. As a result, the performance of deep learning neural networks has seen remarkable improvements [33]. These methods require offline training, which requires a large amount of data, and then can be implemented online to remove artifacts. Due to this first training step, these methods are not usually utilized in the context of neurophysiological signals, in which there is usually no access to a large amount of data. However, there are some interesting works regarding removing ocular artifacts from EEG signals using deep learning-based methods [4,5,6].

As previously mentioned, these techniques are highly diverse and cannot be summarized within a single set of guidelines. In fact, depending on the network architecture, the activation and cost function, and the training methodology, deep learning techniques can significantly vary, encompassing different models. In this regard, Convolutional Neural Networks (CNNs) have been successfully employed for this task. By leveraging their capacity to capture spatial hierarchies of features, CNNs can effectively identify patterns associated with ocular artifacts in the EEG signal. For instance, Deep Convolutional Autoencoders (CAEs) have been utilized to learn compressed representations of clean EEG signals, enabling them to reconstruct clean signals from contaminated input, thereby removing artifacts. Recurrent Neural Networks (RNNs), particularly Long Short-Term Memory (LSTM) networks, have also proven effective in ocular artifact correction. The inherent ability of RNNs to model temporal dependencies makes them adept at capturing the evolving patterns of eye movements reflected in the EEG. LSTMs, with their specialized memory cells, excel at learning long-range dependencies, further enhancing their ability to predict clean EEG segments from contaminated data. Finally, Generative Adversarial Networks (GANs) present another innovative approach to artifact correction. Through an adversarial training process between a generator and a discriminator, GANs can produce highly realistic, clean EEG segments, capturing subtle nuances in the data. This approach holds significant promise for removing complex and diverse ocular artifacts, as demonstrated by frameworks like EEGANet [34].

Regardless of the specific deep learning architecture employed, meticulous data preprocessing, feature extraction, and hyperparameter tuning are critical factors in achieving optimal artifact correction performance. It is crucial to evaluate the efficacy of these methods using appropriate metrics, such as signal-to-noise ratio (SNR), mean squared error (MSE), or correlation coefficient.

For the sake of completeness and to provide a guideline for those interested in studying these algorithms, even though they are less commonly used in neurophysiological contexts, the following process outlines the general steps typically followed in these algorithms. Specifically, this process reflects part of the methodology implemented by Banghua Yang et al. [5].

1. Offline step. The offline step consists of two sub-steps, focusing on extracting the training dataset from the EEG signal. Once the training dataset is created, the model is trained accordingly.
  - Normalizing data and building the training dataset. In this step, the EEG signal is normalized, and the training dataset is built from the raw EEG data. Artifactual samples are removed using statistical thresholds, resulting in a clean EEG dataset without artifacts.
  - Training the chosen deep learning model on the clean data. After this step, the model will need to tune its own parameters and will be capable of recognizing features attributed to uncontaminated EEG.
2. Online step. The online step is the real cleaning process of the algorithm. In this step, the EEG signal is normalized, and the training dataset is built from the raw EEG data. Artifactual samples are removed using statistical thresholds, resulting in a clean EEG dataset without artifacts.

As there is no one classical way to implement these methods, there is no toolbox already open for suggestion. However, usually, machine learning and deep learning applications are implemented in Python using the *numpy* and *panda* libraries.

## 4. Discussion

The methods described in this manuscript for correcting ocular artifacts in EEG signals each have distinct advantages and limitations, making them suitable for different experimental settings and research objectives.

In the following subparagraph, a description of the pros and cons of each method is provided.

### 4.1. Regression-Based Methods

- **Pros**: Regression-based methods are simple and effective when an external template, such as an EOG channel, is available. They can be implemented easily with a small number of EEG channels and are particularly useful for real-time applications.
- **Cons**: The main limitation is that they rely heavily on the quality of the template. If the template is noisy or not perfectly aligned with the artifact in the EEG, the correction may be inaccurate. Additionally, these methods might not fully eliminate artifacts, especially when the EOG signal is strongly correlated with the EEG.
- **Best use cases**: These methods are most effective in controlled laboratory environments where EOG recordings are available, and the primary concern is the removal of blink artifacts with minimal computational complexity. Additionally, the evolutions of such methods, such as the one provided by Reblinca [16], could be indicated for out-of-the-lab application [35,36,37,38,39], where EEG data collection from the frontal or anterofrontal channels is possible.

### 4.2. Independent Component Analysis (ICA)

- **Pros**: ICA is a powerful technique for decomposing EEG signals into independent sources, allowing for precise identification and removal of ocular artifacts without needing additional channels. It is particularly effective in separating overlapping artifacts and neural activity.
- **Cons**: ICA requires a relatively large number of EEG channels to be effective, and its success depends on the quality of the decomposition. It also assumes that the sources are statistically independent, which may not always hold true. Additionally, it is computationally intensive and not ideal for real-time processing.
- **Best use cases**: ICA is best suited for offline analysis in studies with high-density EEG setups, where the goal is to achieve a clean separation of neural and artifact signals for in-depth analysis. In terms of experimental settings, it appears to be clear that the ICA-based techniques are the most indicated when the EEG data collection is performed in laboratory settings [40,41,42,43,44].

### 4.3. Artifact Subspace Reconstruction (ASR)

- **Pros**: ASR is highly effective at removing a wide range of artifacts by reconstructing the EEG signal from a subspace that excludes the contaminated components. It is adaptive and can be used both online and offline, making it versatile.
- **Cons**: ASR’s effectiveness depends on the quality of the initial calibration data. Poor calibration can lead to overcorrection, where some neural signals might be mistakenly removed. It also requires more computational resources compared to simpler methods, like regression.
- **Best use cases**: ASR is ideal for both online and offline applications [1,45,46], particularly in environments where artifacts are frequent and varied, such as in mobile EEG studies [2,47,48,49] or complex experimental setups.

### 4.4. Deep Learning-Based Algorithms

- **Pros**: Deep learning models, particularly Convolutional Neural Networks (CNNs) and Recurrent Neural Networks (RNNs), can outperform traditional methods like ICA and regression-based techniques in terms of accuracy. These models can automatically learn intricate, non-linear patterns in EEG signals, which allows them to separate ocular artifacts from neural activity with higher precision. Such methods do not need any manual and/or visual intervention, like ICA, and they are robust in terms of handling large and complex datasets, making them ideal for high-density EEG data. Furthermore, such models can generalize well to new and unseen datasets. This makes these methods highly adaptable across different individuals, experimental conditions, and EEG system configurations.
- **Cons**: One of the possible limitations of deep learning-based methods consists of the need for large, annotated training datasets to achieve high performance. Moreover, such models are computationally expensive, especially during the training phase. A further limitation to consider corresponds to the overfitting risk, which could occur if the training dataset is not large or diverse enough. Finally, one of the major concerns with deep learning models is the “black box” nature of neural networks. Unlike traditional methods like ICA, which offer interpretable components corresponding to underlying brain processes, deep learning models do not easily offer insights into *how* the correction is being performed.
- **Best use cases**: As suggested by the positive aspects associated with these methods, deep learning approaches are ideal for scenarios where large-scale datasets with high-density EEG are available [49,50,51,52]. Therefore, they could be implemented for real-time artifact correction in BCIs [53,54,55,56]. In these cases, the need for immediate feedback requires robust artifact detection and removal, which deep learning methods can provide, especially when artifact patterns are non-linear or difficult to model with traditional methods. In parallel, these approaches are particularly indicated when dealing with EEG data collected through wearable and mobile systems. As this kind of equipment becomes more prevalent for in-field research, deep learning methods offer the potential for on-device, real-time processing.

## 5. Conclusions

The correction of ocular artifacts in EEG signals remains a critical challenge, especially as the applications of wearable EEG systems in real-world environments expand. This tutorial provides a detailed exploration of various state-of-the-art methods for identifying and correcting ocular artifacts, emphasizing their respective advantages and limitations. By examining techniques such as regression-based methods, Independent Component Analysis (ICA), Artifact Subspace Reconstruction (ASR), and deep learning approaches, we outline how each of these methods can be applied based on the specific context of the EEG recording.

Among the presented techniques, regression-based methods offer simplicity and real-time applicability, while ICA provides high accuracy for high-density EEG setups, but requires significant computational resources. ASR stands out for its adaptability in both online and offline applications, especially in mobile EEG studies. Deep learning methods, while highly promising, require large datasets for training and may not be as widely accessible for all neurophysiological studies.

The tutorial has also highlighted the importance of selecting the appropriate method based on the study’s experimental setup, the available EEG channels, and the computational resources. As wearable EEG systems and real-time applications continue to evolve, the need for adaptable and efficient artifact correction methods will grow.

In summary, the choice of artifact correction method should be guided by the specific needs of the research or application at hand. This tutorial provides a foundational understanding that researchers can build upon to select the most appropriate techniques for their studies. As the field progresses, the development and validation of new methods will be crucial in ensuring that EEG data remain a reliable tool for understanding brain function in increasingly complex and dynamic environments.

## Data Availability

Not applicable.
